# Behead and live long or the tale of cathepsin L

**DOI:** 10.1002/yea.3286

**Published:** 2017-11-29

**Authors:** Maria Karolin Streubel, Johannes Bischof, Richard Weiss, Jutta Duschl, Wolfgang Liedl, Herbert Wimmer, Michael Breitenbach, Manuela Weber, Florian Geltinger, Klaus Richter, Mark Rinnerthaler

**Affiliations:** ^1^ Department of Cell Biology and Physiology, Division of Genetics University of Salzburg Salzburg Austria; ^2^ Department of Molecular Biology University of Salzburg Salzburg Austria

**Keywords:** aging reporter, amyloid fibrils, cathepsin L, cysteine protease, flatworm, prions, protein aggregates, replicative aging

## Abstract

In recent decades Saccharomyces cerevisiae has proven to be one of the most valuable model organisms of aging research. Pathways such as autophagy or the effect of substances like resveratrol and spermidine that prolong the replicative as well as chronological lifespan of cells were described for the first time in S. cerevisiae. In this study we describe the establishment of an aging reporter that allows a reliable and relative quick screening of substances and genes that have an impact on the replicative lifespan. A cDNA library of the flatworm Dugesia tigrina that can be immortalized by beheading was screened using this aging reporter. Of all the flatworm genes, only one could be identified that significantly increased the replicative lifespan of S.cerevisiae. This gene is the cysteine protease cathepsin L that was sequenced for the first time in this study. We were able to show that this protease has the capability to degrade such proteins as the yeast Sup35 protein or the human α‐synuclein protein in yeast cells that are both capable of forming cytosolic toxic aggregates. The degradation of these proteins by cathepsin L prevents the formation of these unfolded protein aggregates and this seems to be responsible for the increase in replicative lifespan.

## 1 INTRODUCTION

During the last decade it turned out that aging mechanisms and pathways that lead to longevity are evolutionarily conserved (Wasko & Kaeberlein, 2014). One model organism that has driven the advances in the field of aging research is Saccharomyces cerevisiae, but other model organisms such as Mus musculus, Drosophila melanogaster and Caenorhabditis elegans also contributed to the understanding of aging.

The advantages of this important single cell eukaryote are its being a powerful genetic system as well as the possibility to modulate mitochondrial respiration (and in this way to modulate the production of reactive oxygen species). Furthermore there is the possibility of studying two forms of aging processes [chronological and mother‐cell specific (replicative) aging], which is an additional advantage. Chronological aging describes the loss of viability during stationary phase and resembles in this way the aging processes in non‐dividing, post‐mitotic, differentiated human cells. The other aging process is called mother cell‐specific aging and is based on the fact that the mother cell ages, whereas the daughter cell rejuvenates itself by a mechanism that is still not fully understood (Laun, Rinnerthaler, Bogengruber, Heeren, & Breitenbach, 2006). For this mechanism an asymmetric inheritance of organelles (Klinger et al., 2010) and/or damaged proteins is the focus of research (Liu et al., 2010). Comparable asymmetric cell divisions can also be seen in human stem cells that are delivering differentiated cells and are the main determinant of the Hayflick limit (although some data indicate that the Hayflick limit is strongly influenced by oxygen toxification; Di Micco et al., 2008).

The aging mechanisms that were mainly or partially unravelled in yeast cells are manifold (some are still controversially discussed): caloric restriction (Lin, Defossez, & Guarente, 2000); nutrient sensing via the cAMP‐dependent protein kinase A pathway (Wasko & Kaeberlein, 2014); stimulation of autophagy (Takeshige, Baba, Tsuboi, Noda, & Ohsumi, 1992); mTOR pathway (Kaeberlein et al., 2005); histone deacetylation via sirtuins (Wood et al., 2004); reduced ribosomal activity (Chiocchetti et al., 2007); apoptosis (Laun et al., 2001); and increase in protein turnover (Kruegel et al., 2011). All of the anti‐aging drug interventions that will most probably increase the healthy lifespan of humans were first identified and described in yeast cells: rapamycin (Powers, Kaeberlein, Caldwell, Kennedy, & Fields, 2006), resveratrol (Howitz et al., 2014) and spermidine (Eisenberg et al., 2009).

In the current study we try to combine the power of the highly developed yeast genetics with the power of a spectacular model organism that is still not in the focus of research. Flatworms are known to have an incredibly high regeneration capacity. *Macrostomum lignano*, a marine, free‐living flatworm, can be cut into pieces and the head can fully regenerate the complete posterior part of the body. The only body part that cannot be regenerated is the head. Beheading and recultivation of the head is even having a positive side effect: *M. lignano* lives for about 10 months, but Egger et al. were able to demonstrate that a series of regenerations of the flatworms’ body more than doubled the lifespan of this marvelous organism. It is quite possible that this organism is made immortal by beheading (Egger, Ladurner, Nimeth, Gschwentner, & Rieger, 2006; Egger, Gschwentner, & Rieger, 2007).

As will be demonstrated below, we successfully established an aging reporter in S. cerevisiae that enables us to easily measure the replicative lifespan of yeast cells. In this way we can screen for substances and genes that are capable of prolonging the lifespan. In the present study we combined our aging reporter with a cDNA library from Dugesia tigrina (another tubellaria congeneric to *M. lignano)*. By testing all flatworm genes in S. cerevisiae we identified the gene cathepsin L‐like that greatly improves the replicative lifespan of yeast cells. Owing to the high sequence identity between cathepsin L‐like from *Dugesia* and its homologue in *Hydra*, this finding could contribute to the understanding of *Hydra*’s immortality.

## 2 MATERIAL AND METHODS

### 2.1 Yeast strains and media

The S. cerevisiae strains BY4741 (*MATa his3*Δ*1 leu2*Δ*0 met15*Δ*0 ura3*Δ*0*) and BY4742 (*MATα his3*Δ*1 leu2*Δ*0 lys2*Δ*0 ura3*Δ*0*) and modifications to them were used for all of the experiments. The cells were either cultivated in complex medium [YPD; 1% (w/v) yeast extract, 2% (w/v) peptone and 2% (w/v) d‐glucose] or synthetic complete glucose medium [SC‐glucose; 2% (w/v) d‐glucose, 0.17% (w/v) yeast nitrogen base without amino acids, 0.5% ammonium sulphate and 10 mL of complete dropout mixture (0.2% Arg, 0.1% His, 0.6% Ile, 0.6% Leu, 0.4% Lys, 0.1% Met, 0.6% Phe, 0.5% Thr, 0.4% Trp, 0.1% Ade, 0.4% Ura, 0.5% Tyr) per litre]. Respective solid media were prepared by adding 2% (w/v) agar. By leaving out the appropriate amino acids from the synthetic complete glucose medium, a selection for plasmids was achieved.

### 2.2 Construction of the aging reporter

The HO promoter was PCR amplified from genomic DNA obtained from the strain K6001 (Jarolim et al., 2004) using primers with the following sequences: 5′‐AACTG CAGAT AGAAA ACATA TGCTA TAAG‐3′; 5′‐ACGCG TCGAC TTTAA AGTAT AGATA GAA‐3′. The resulting ~2.5 kb fragment was digested with SalI and PstI and was cloned into the vector YCplac111(Gietz & Sugino, 1988). eGFP was excised from the vector pUG35 (Niedenthal, Riles, Johnston, & Hegemann, 1996) with the enzymes SmaI and XbaI and cloned into the vector YCplAc111‐HOprom. All restriction enzymes were provided by Promega (Mannheim, Germany), NEB (Ipswich, USA) or Thermo Scientific (Waltham, MA, USA) and all of the constructs were sequenced by Eurofins‐MWG‐OPERON (Ebersberg, Germany).

### 2.3 Yeast transformation

Exponentially growing cells (OD_600_ of ~0.8; ~5 × 10^8^ cells) were centrifuged at 3500 ***g*** for 3 min, washed with water and resuspended in 200 μL LiAc/TE (100 mm LiAc, 10 mm Tris, 1 mm EDTA, pH 8.0). A 50 μL aliquot of this cell paste was mixed with 300 μL of LPT (100 mm LiAc, 10 mm Tris, 1 mm EDTA, ph 8.0, 50% PEG 3350), 5 μL of salmon sperm (10 μg/μL) and 5 μg of plasmid DNA. After a 30 min incubation at room temperature, 40 μL DMSO was added and a heat shock (42°C, 15 min) was applied. The cells were then washed and regenerated for 2 h at 28°C in YPD. Finally the cells were plated on the specific selective medium plates.

### 2.4 Elutriation

The elutriation was performed as described in Klinger et al. (2010). BY4741 YCplac111‐HOprom‐GFP p416GPD or BY4741 YCplac111HOprom‐GFP p416GPD‐cDNA (D. tigrina) was grown overnight in SC‐Leu‐Ura medium (buffered with 100 mm BES at pH 7.5 to improve GFP fluorescence). This culture was then diluted to an OD_600_ 0.1 in 500 mL SC‐Leu‐Ura and grown for 2 days to stationary phase in SC‐Leu‐Ura. The elutriation was performed with the Beckman elutriation system and rotor JE‐6B with a standard elutriation chamber. Prior to elutriation cells were centrifuged at 3000 rpm for 10 min, resuspended in 10 mL 1 × PBS and sonicated to separate mother from daughter cells. The cells were then loaded into the elutriation chamber with a rotor speed of 2700 rpm and a flow rate of 10 mL/min. Reduction of the rotor speed to 1350 rpm yielded fractions III–V and these three fractions were inoculated again in SC‐Leu‐Ura to generate cells of higher age. After 2 days a second elutriation was performed with altered parameters. The cells were loaded at 3200 rpm and a reduction of the rotor speed to 2700 rpm yielded fraction II. To obtain fraction V cells the rotor speed was decreased to 2000 rpm to get rid of fractions III and IV, only retaining fraction V. Setting the rotor speed to 1350 rpm led to fraction V. If necessary, 10 μm resveratrol was added to the cultures before and after the first elutriation.

### 2.5 Construction of the D. tigrina cDNA library

A 50 mg aliquot of D. tigrina was resuspended in 350 μL buffer RLT (Qiagen, Hilden, Germany), homogenized using a potter homogenizer, and RNA was isolated using the RNeasy® Mini Kit (Qiagen). cDNA synthesis was performed with the SMARter™ RACE cDNA amplification kit (Clonetech, Mountain View, USA) according the manufacturer's instructions. In the process the cDNA was tagged at the 5′ site with the sequence 5′‐AAGCAGTGGTATCAACGCAGAGTAC‐3′ and at 3′ site with the sequence 5′‐GGCCGGAGAGAGAGACTGCAGACTCGAGA‐3′. The resulting cDNA was PCR amplified with the LongAmp® TAQ DNA polymerase (NEB, Ipswich, USA) using the primers 5′‐ACATA ACTAA TTACA TGACA CTGCA GACTC GAGAT T‐3′ and 5′‐GGATT CTAGA ACTAG TGGAT CCTGG TATCA ACGCA GAGTA‐3′. The PCR products were cloned into the vector p416GPD that was linearized with BamHI and XhoI using the Gibson Assembly ® Cloning Kit (NEB, Ipswich, USA).

### 2.6 Cloning of cathepsin L and human *α*‐synculein

Cathepsin L was PCR amplified with the Phusion® High‐Fidelity DNA Polymerase (NEB, Ipswich, MA, USA) from p416GPD‐cathpsin L using the primers 5′‐GCGGA TCCAT GGCAC CAAAA AATAT GGGGA AA‐3′ and 5′‐TTCCG GAATT CAACC AAGGG ATAGC TGGCC A‐3′. The PCR product was cloned into the vector pUG35 using the enzymes BamHI and EcoRI.

Cathepsin L was also cloned into the expression vector pESC‐HIS by transferring it from p416‐GPD using the restriction enzymes BamHI and XhoI. *α*‐Synculein was PCR amplified from human cDNA (Rinnerthaler et al., 2013) using the primers 5′‐TCTAG AATGG ATGTA TTCAT GAAAG GAC‐3′ and 5′‐ACGCG TCGAC GGCTT CAGGT TCGTA GTCTT‐3′. Using the enzymes XbaI and SalI this human gene was cloned into the vector pUG35, creating a GFP tagged version.

The RFP tagged version of cathepsin L was cloned into the vector pESC‐HIS using Gibson Assembly; pESC‐HIS was linearized using BamHI and EcoRI, whereas RFP (5′‐TCCCT TGGTT ATGAC CATGA TTACG CCAAG CG‐3′ and 5′‐CTATA GTGAG TCGTA TTACG TTAGG CGCCG GTGGA GTG‐3′) and cathspin L (5′‐GTCAA GGAGA AAAAA CCCCG GATCC ATGGC ACCAA AAAAT ATGGG‐3′ and 5′‐TCATG GTCAT AACCA AGGGA TAGCT GGC‐3′) were PCR amplified. RFP and cathepsin L were cloned at the same time in a one‐tube reaction into the vector.

Cathepsin L‐RFP was recloned into p416GPD using Gibson Assembly. The vector p416GFP was linearized with the enzymes X and Y, and cathepsin L‐RFP was PCR amplified (5′‐GACGG ATTCT AGAAC TAGTG GATCC ATGGC ACCAA AAAAT ATGGG GAAAT TACCT GGCAC G‐3′ and 5′‐CGAAT TCCTG CAGCC CGGGG GGCGC CGGTG GAGTG GCG‐3)′.

### 2.7 Mutation of cathepsin L

In *Dugesia* cathepsin L a cysteine was replaced with a serine using mutagenic primers. Two PCRs were performed using p416GPD as a matrix. For the first PCR 5′‐TGAAC CGCAT TGTTC TTGAT TTTTC‐3′ and 5′‐CAAGG GATAT ATTAA AATGT CAAAG GATG‐3′ were used as primers. For the second PCR the mutagenic primers 5′‐ATCAA GAACA ATGCG GTTCA *A*GTTG GTCAT TTTCA GCAAC AGG‐3′ (the mutagenic nucleotide is flanked by asterisks) and 5′‐ACATT TTAAT ATATC CCTTG ATACC CCAAC CAGTT CCCCA AC‐3′ weew used. The two PCR products were circularized using the Gibson Assembly® Cloning Kit (NEB, Ipswich, USA). The replacement was confirmed via sequencing at Eurofins‐MWG‐OPERON (Ebersberg, Germany).

### 2.8 Isolation of plasmid from S. cerevisiae

Yeast cells were resuspended in 500 μL SCE (1 m sorbitol, 20 mm EDTA, 10 mm Na‐citrate pH 7); 40 μL zymolyase (10 mg/mL) was added and incubated for 60 min at 37°C. The cells were lysed by addition of 60 μL 10% SDS and an incubation at 65°C for 30 min. After addition of 200 μL 5 m potassium acetate pH 5.0 the cell lysate was incubated on ice for 1 h.

The supernatant, after a centrifugation for 5 min at 14,000 rpm, was mixed with isopropanol and the DNA was precipitated at −20°C. The DNA was pelleted for 15 min at 14,000 rpm, washed once with 70% ethanol and dried in the desiccator. The DNA pellet was dissolved in 20 μL H_2_O and directly transformed into competent E. coli cells. The plasmid DNA was then isolated from E. coli and the identity of the inserts was determined by sequencing at Eurofins‐MWG‐OPERON (Ebersberg, Germany).

### 2.9 Replicative lifespan

The replicative lifespan was analysed as described previously (Pichova, Vondrakova, & Breitenbach, 1997). For the determination of the replicative lifespan a Singer MSM microscope and micromanipulator were used. The lifespan study was performed with at least 45 virgin cells and the daughters produced were counted and removed. During day the plates were incubated at 28°C, and during night at 4°C. The lifespans were performed on either SC‐glucose media or SC‐Ura‐Leu media. The significances in lifespan differences were calculated with log‐rank statistics at the 98% confidence level using an available online tool (http://www.evanmiller.org/ab‐testing/survival‐curves.html).

### 2.10 ImmunoBlot

Western Blot analysis was performed as described in Bischof et al. (2017). Cells were broken by vortexing in the presence of glass beads (diameter 0.25–0.5 mm). Equal protein amounts were loaded on a 15% SDS‐PAGE gel. Proteins were blotted on a protran BA85 nitrocellulose membrane (Schleicher & Schuell, Germany) with 250 mA for 90 min at RT. The membrane was blocked with MTBS‐T (25 mm Tris pH 7.6, 137 mm NaCl, 0.1% Tween 20, 5% milk powder) for 90 min at RT and washed for 30 min with TBS‐T. The primary anti‐GFP‐antibody (SC‐9996; Santa Cruz Biotechnology, Santa Cruz, CA, USA) was diluted in MTBS‐T (1:1000) and incubated overnight at 4°C under constant shaking. The blot was washed three times with TBS‐T (3 × 10 min at RT) and incubated for 2 h at RT with a horse anti‐mouse IgG antibody [no. 7076; NEB/Cell Signaling (Ipswich, USA)]. The secondary antibody was diluted in MTBS‐T 1:3000. Detection was carried out with the Pierce (Thermo Fisher Scientific, Waltham, MA, USA) ECL western blotting substrate according to the manufacturer's instructions.

### 2.11 Flow cytometry and cell sorting

GFP expression in cells transfected with the various reporter plasmids was analysed by flow cytometry using a FACS Canto II cytometer (BD Biosciences). Briefly, viable cells were first gated based on their forward (FSC) and side scatter (SCC) properties. Cell doublets were excluded using FSC‐width vs. FSC‐height. Old cells expressing high levels of GFP were sorted from old cells with low GFP fluorescence (fraction V elutriation) using a FACS Aria III high speed cell sorter (BD Biosciences) with a 70 μm nozzle. Sorting was performed at 4°C. The cut‐off for GFP‐high cells was defined based on cells transformed with an empty control plasmid.

### 2.12 Fluorescence microscopy

The fusion proteins (either GFP or RFP fusions) were analysed with a × 100 objective (NA  =  1.4) using either a Carl Zeiss AG Axioscope (Oberkochen, Germany) or a Nikon (Tokyo, Japan) Eclipse Ni‐U equipped with a DS‐Fi2 digital camera. The grade of co‐localization was quantified as described in Rinnerthaler et al. (Rinnerthaler et al., 2013) using the Co‐localization Finder plugin as part of NIH ImageJ software.

### 2.13 GFP quantification

An aliquot of 5 × 10^6^ cells in a volume of 200 μL PBS was pipetted in a black microwell plate (Nunc). The fluorescence was then measured in an Anthos Zenyth 3100 (Anthos Labtec Instruments) plate reader with an excitation wavelength of 485 nm and an emission wavelength of 595 nm.

### 2.14 RNA isolation

An aliquot of 5 × 10^9^ cells was resuspended in Peqlab TriFast (VWR) and broken by vortexing. Total RNA was isolated according to the manufacturer's instructions.

### 2.15 cDNA synthesis

cDNA was synthesized as described in Rinnerthaler et al. (2013). A 0.5 μg aliquot of RNA was reversed transcribed using 2 μg oligo‐dT‐primer and the MMLV High Performance Reverse Transcriptase (Epicenter, Madison, WI, USA) according to the manufacturer's instructions.

### 2.16 Real‐time PCR

The following primers were ordered from Sigma‐Aldrich Co. LLC: TDH3 sense – 5′‐TTGACGGTCCATCCCACAAG‐3′; TDH3 antisense – 5′‐CGACG GTTGG GACTC TGAAA‐3′; SUP35 sense – 5′‐CCGGC CCAAC TCTGT TAGAA‐3′; SUP35 antisense – 5′‐CCACA GCGGT TTTGT TAGGC‐3′. RT‐PCR analysis was performed using a Rotor Gene Q (Qiagen) and the GoTaq qPCR Master Mix (Promega Madison, WI, USA). The following cycling conditions were used: 95°C 240 s; 40 cycles of 95°C 10 s, 65°C 15 s, 72°C 15 s and 72°C for 300 s. TDH3 was chosen as a reference for normalization. The fold change was calculated according to the formula 2^−(ΔΔ*Ct*)^.

### 2.17 ThioS staining

Yeast cells were stained as described in Kimura, Koitabashi, and Fujita (2003) and Lee, Shin, Choi, Lee, and Lee (2002). After formaldehyde fixation (3% formaldehyde in PBS for 30 min at room temperature) the cells were resuspended in 1 × PBS containing 0.05% Thioflavin S and incubated for 15 min at room temperature. The cells were then washed three times with 1 × PBS and analysed via fluorescence‐microscopy.

## 3 RESULTS AND DISCUSSION

### 3.1 Aging reporter

**Figure 1:**
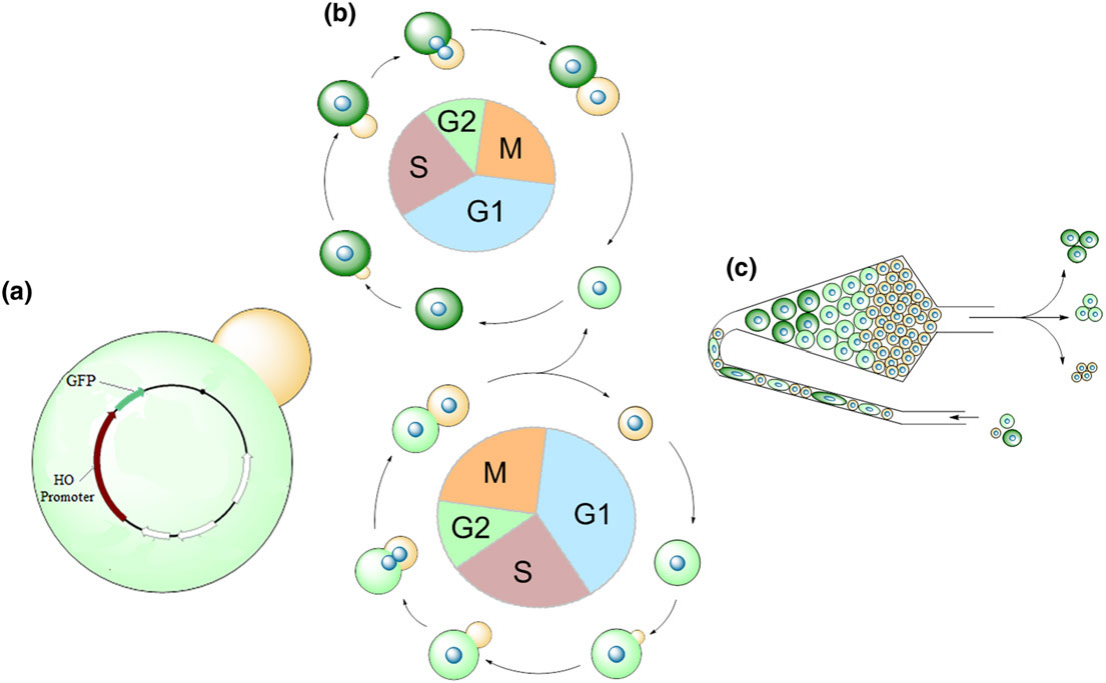
Overview of the aging reporter. Yeast cells are transformed with the plasmid YCplac111 harbouring the GFP molecule under control of the HO endonuclease promoter (a). Each time the yeast cells divide, GFP molecules are produced at the transition from the G1 to S‐phase. In this way replicative aged yeast cells have a much brighter GFP fluorescence than young cells (b). Using elutriation centrifugation old cells (bright GFP fluorescence) are separated from young cells (low GFP fluorescence). The GFP fluorescence gives a direct feedback to the age of yeast cells and can be used to screen for substances as well as genes that prolong the replicative lifespan (c)

**Figure 2:**
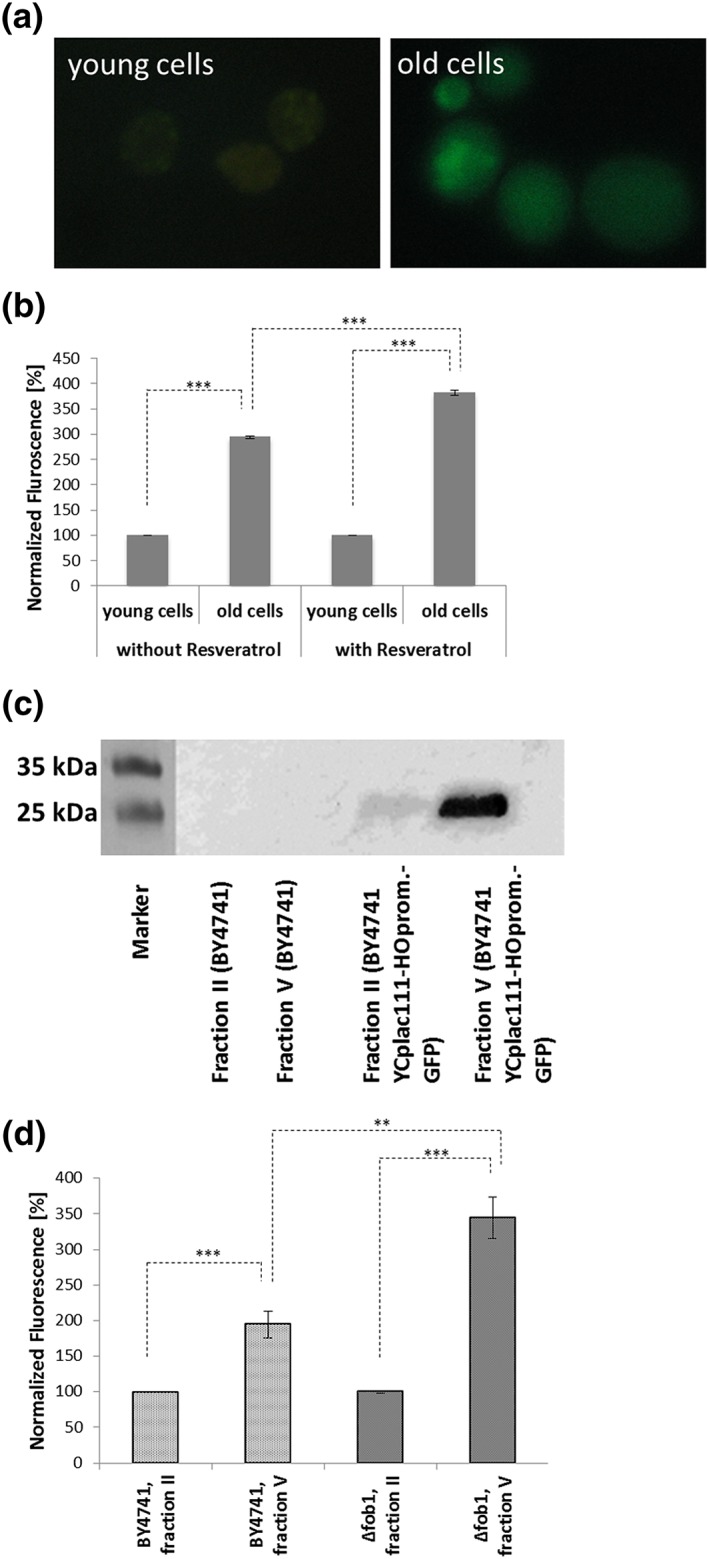
Verification of the aging reporter. The strain BY4741 YCplac111‐HOprom‐GFP was elutriated and in this way young cells (fraction II) separated from old cells (fraction V). The young cells only showed a very faint GFP signal, whereas the old cells showed a clear increase in fluorescence (a). Western blot analysis of the strains BY4741 YCPlac111‐HOprom and BY4741 YCplac111‐HOprom‐GFP after elutriation using an anti GFP‐antibody. Homogenates of fraction II/young cells (BY4741 YCPlac111‐HOprom and BY4741 YCplac111‐HOprom‐GFP) and fraction V/old cells (BY4741 YCPlac111‐HOprom and BY4741 YCplac111‐HOprom‐GFP) were loaded onto the gel. In case of the wild‐type no GFP was detectable, whereas in case of the BY4741 YCplac111‐HOprom‐GFP strain a much stronger signal was detectable in fraction V (b). Fluorometric measurement of BY4741 YCplac111‐HOprom‐GFP cells after elutriation. In case of a treatment with 10 μm resveratrol the old cells had a statistically significant (student *t*‐test; *p*‐value = 0.0001) higher GFP fluorescence than untreated cells (c). Fluorometric measurement of the strains BY4741 YCplac111‐HOprom‐GFP and *fob1Δ* YCplac111‐HOprom‐GFP. Deletion of *FOB1* leads to an increase of GFP fluorescence in fraction V cells, indicating an increased replicative lifespan [Colour figure can be viewed at wileyonlinelibrary.com]

In a first step a ‘replicative aging’ reporter was created that allows for the screening of life‐prolonging drugs and longevity genes. This reporter is based on the *HO* endonuclease that is cleaving DNA at the *MAT* locus, a prerequisite for the mating type switch. HO is tightly repressed during the whole cell cycle but is expressed at the G1/S transition (Jarolim et al., 2004). Both GFP and the promoter of the *HO* gene were cloned into the centromeric vector YCPlac111 (Figure 1). The idea is that each time yeast cells divide they express GFP (before they start to replicate), rendering yeast cells that get more and more fluorescent while aging. This reporter was transformed into the BY4741 background yielding yeast cells that only showed a very faint GFP signal. One of the hallmarks of aging in yeast cells is a dramatic increase in cell volume and this phenotype can be used to isolate/enrich old yeast cells. The proportion of old cells in a dividing culture is usually very low (<0.3%). By using elutriation, a counterflow centrifugation technique, young cells (fraction II) were separated from old cells (fraction V). In fraction II all the cells are nearly devoid of a GFP signal, whereas in fraction V that is enriched in old cells many cells show a robust green fluorescence (see Figure 2a). Using the Anthos Zenyth 3100 fluorimeter the difference in green fluorescence was quantified. Old yeast cells showed a ~2.9‐fold increase in fluorescence intensity (Figure 2b). To exclude possible side effects of auto‐fluorescence, a Western Blot analysis using a GFP‐antibody was performed (Figure 2c). Two strains were analysed: BY4741 YCplac111‐HOprom. (control) and YCplac111‐HOprom.‐GFP (aging reporter). Fractions II and V of the control strain yielded no GFP‐specific signal on the western blot, whereas the young and old cells of the strain harbouring the aging reporter showed a clear signal at the height of ~30 kDa (predicted MW for GFP was 28 kDa). In the case of fraction V cells the signal is much stronger (6.2‐fold, as calculated with ImageJ), confirming the accumulation of GFP in old cells.

Further tests with this reporter were performed with the drug resveratrol and a deletion strain of *FOB1*. Resveratrol directly (Howitz et al., 2003) or indirectly (Price et al., 2012) activates sirtuins and promotes gene silencing and autophagy (Park et al., 2016), resulting in an increase in the replicative lifespan in yeast cells. A yeast culture harbouring the aging reporter was grown in the presence of 10 μm resveratrol and after two cycles of elutriation (for details see the ‘Material and methods’ section) the fluorescence in the old and young fraction was quantified. In contrast to untreated cells, the GFP fluorescence in old cells increased more than 3.8‐fold when comparing fraction V with fraction II, thus proving the feasibility of this reporter system (Figure 2b). Finally the aging reporter was tested in a strain deleted for *FOB1*. *Δfob1* was published to reduce the number of extrachromosomal rDNA circles, thus increasing the replicative lifespan of yeast cells (Defossez et al., 1999). This strain was transformed with YCplac111‐HOprom.‐GFP. In fact the GFP fluorescence of old cells increased from ~3‐fold (comparing young and old cells) in the control strain to more than 3.6‐fold in the *Δfob1* strain (Figure 2d).

### 3.2 D. tigrina In S. cerevisiae

**Figure 3:**
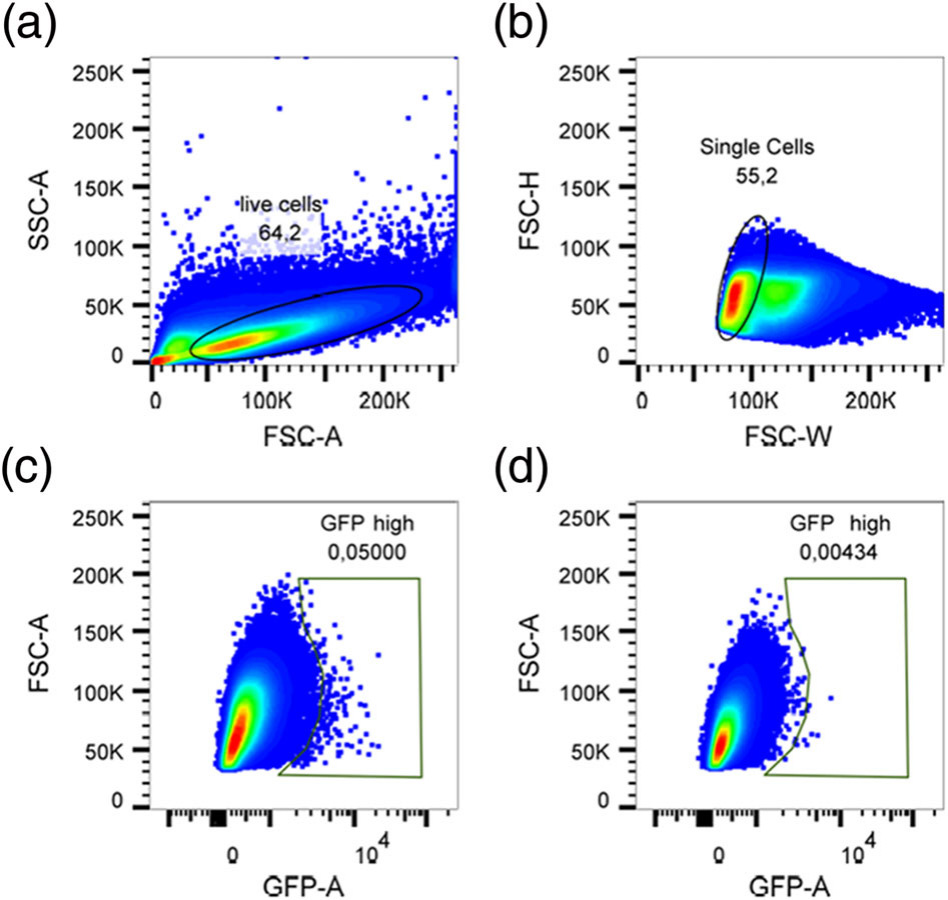
Sorting of GFP high cells from yeast cells transformed with an aging reporter. Cells were first gated on a FSC/SSC plot to exclude dead cells and debris (a). Subsequently, single cells were gated based on FSC‐H/FSC‐W (b). Yeast cells transformed with the GFP‐reporter plasmid showed 0.05% of GFP‐high cells (c), a 10‐fold higher amount compared with yeast cells transformed with the control plasmid (d) [Colour figure can be viewed at wileyonlinelibrary.com]

**Figure 4:**
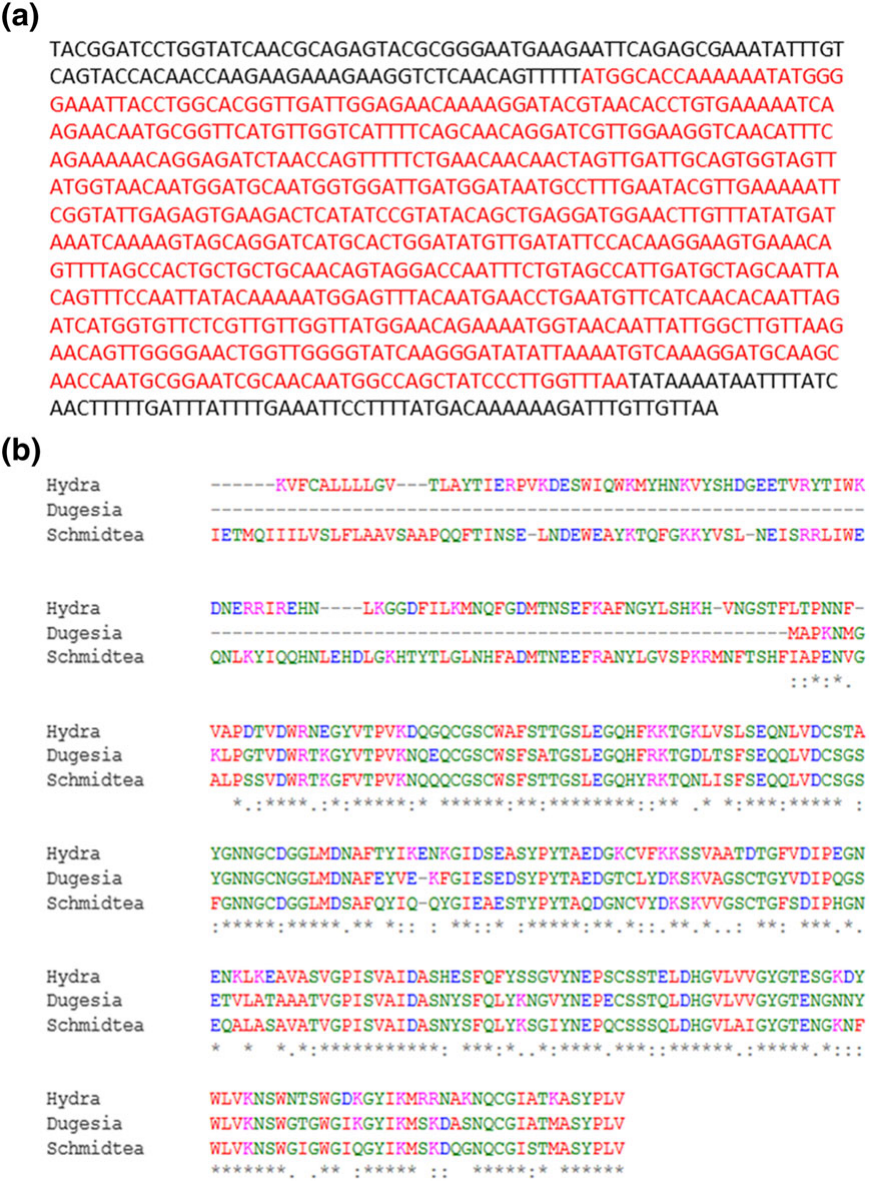
Identification of cathepsin L. the sequence of cathepsin L from Dugesia tigrina is presented (a). A multiple sequence alignment comparing cathepsin L from D. tigrina *, Schmidtea mediterranea* and *Hydra vulgaris* using Clustal omega (http://www.ebi.ac.uk/Tools/msa/clustalo/) was performed. The high similarity between these three proteins is very obvious. An asterix stands for a conserved residues, a colon stands for highly similar residues and a period stands for a weakly similar residues [Colour figure can be viewed at wileyonlinelibrary.com]

After validation of the aging reporter, a D. tigrina cDNA library (see the ‘Materials and methods’ section) in the yeast expression vector p416GPD was created and transformed into the yeast strain BY4741 YCplac111‐HOprom.‐GFP. In this yeast library under control of the GPD (glyceraldehyde‐3‐phosphate dehydrogenase) promoter, flatworm genes will be expressed in a constitutive way. A yeast culture in which the cells express different flatworm genes were elutriated two times, rendering fraction V cells that are enriched in replicatively aged cells. These cells were then analysed by flow cytometry. After gating on live (Figure 3a), single (Figure 3b) cells, a small fraction of cells with a dramatic increase in green fluorescence (Figure 3c) compared with the control (BY4741 YCplac111‐HOprom.‐GFP p416GPD, Figure 3d) could be identified. The cells with the strongest fluorescence (most probably very old cells) were sorted and plated on SC‐Leu‐Ura media. Approximately 100 colonies were processed further. The plasmid DNA was isolated, digested with BamHI and XhoI (to prove the presence of an insert) and sequenced using a plasmid‐specific primer (5′‐AGGTATTGATTGTAATTCTG‐3′). Of all the sequenced genes (all of them sequenced for the first time), only one was overrepresented (25.9% of all clones). The sequence is presented in Figure 4(a). A blast search revealed that this gene is most probably coding for a cathepsin L‐like protein. The closest homologues are found in the freshwater planarian *Schmidtea mediterranea* (78% identical) and *Hydra vulgaris* (72% identical) (Figure 4b).

### 3.3 Cathepsin L in S. cerevisiae

The isolated plasmid, harbouring the cathepsin L‐like gene, was retransformed again into the strain BY4741 YCplac111‐HOprom.‐GFP. After elutriation fraction V cells were analysed with FACS BD Aria III. A robust increase in GFP fluorescence compared with control cells was reproducibly observed (see Figure S1 in the Supporting Information).

**Figure 5:**
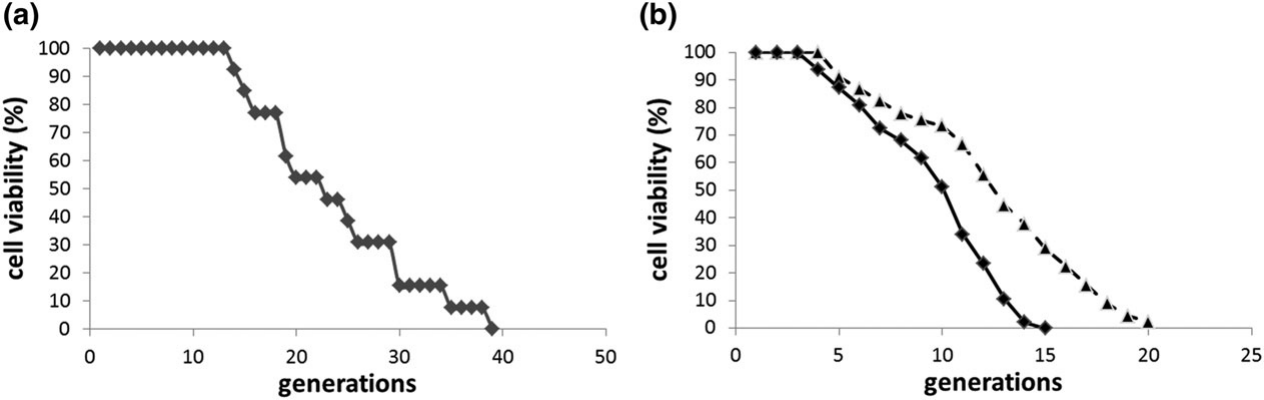
Cathepsin L and the replicative lifespan. A mother cell specific lifespan with the strain BY4741 was performed using a singer micromanipulator (a). Transformation of BY4741 with the two plasmid YCplac111–HOprom.‐GFP p416GPD (continuous line; diamonds) leads to a decreased replicative lifespan that increases greatly and significantly (*p*‐value <0.001) upon expression of cathepsin L (p416GPD‐cathepsin L) (dotted lines; triangles)

The results of the FACS analysis were confirmed with micromanipulation/replicative lifespan analysis as established previously (Kennedy, Austriaco, Zhang, & Guarente, 1995). As a test the BY4741 background harbouring no plasmid was analysed on SC media (Figure 5a). A mean lifespan of 23.1 generations and a median lifespan of 22 generations were observed, fitting well to already published data (Huberts et al., 2014). In the following two strains were analysed: BY4741 YCplac111‐HOprom.‐GFP p416GPD and BY4741 YCplac111‐HOprom.‐GFP p416GPD‐cathepsin L on SC‐Ura‐Leu media. A dramatic drop in the replicative lifespan was observed. The control strain (BY4741 YCplac111‐HOprom.‐GFP p416GPD) showed a mean lifespan of 8.9 generations and a median lifespan of 10 generations. This drop in the production of daughters is attributed to the fact that autonomously replicating sequences on plasmids is known to reduce the lifespan of yeast cells (Falcon & Aris, 2003). The presence of cathepsin L from D. tigrina significantly (*p* < 0.001) increased the mean (11.9 generations) as well median lifespan (12 generations) (Figure 5b).

This ~34% increase in replicative lifespan can most probably be attributed to the endoprotease cathepsin L. In mammals this cysteine cathepsin is primarily found in the lysosome and the extracellular space, but it was also found in the cytoplasm and nucleus and is involved in the terminal degradation of intracellular proteins (Adams‐Cioaba, Krupa, Xu, Mort, & Min, 2011; Sudhan & Siemann, 2015; Sullivan et al., 2009). Among the targets of this protease are collagen, elastin, histone H3, the transcription factor CUX1 and many more (Adams‐Cioaba et al., 2011; Liu et al., 2006). Cathepsin L is also capable of degrading *α*‐synuclein amyloid fibrils that are believed to be a main contributor to Parkinson's disease (McGlinchey & Lee, 2015) and polyglutamine‐containing protein aggregates that cause several neurodegenerative diseases (Bhutani, Piccirillo, Hourez, Venkatraman, & Goldberg, 2012). The alignment (Figure 4b) clearly shows that the *N‐*terminal part in *Dugesia* is missing that is present in nearly all other organisms. Cathepsin L in humans is encoded as a pre‐proenzyme and the *N‐*terminal domain is inhibiting its activity (Coulombe et al., 1996). Because of the absence of this regulatory domain, it has to be concluded that this enzyme is of great importance for D. tigrina and is constitutively active. This leads also to the assumption that the enzyme is active in yeast cells without further regulation.

### 3.4 Cathepsin L and Sup35

**Figure 6:**
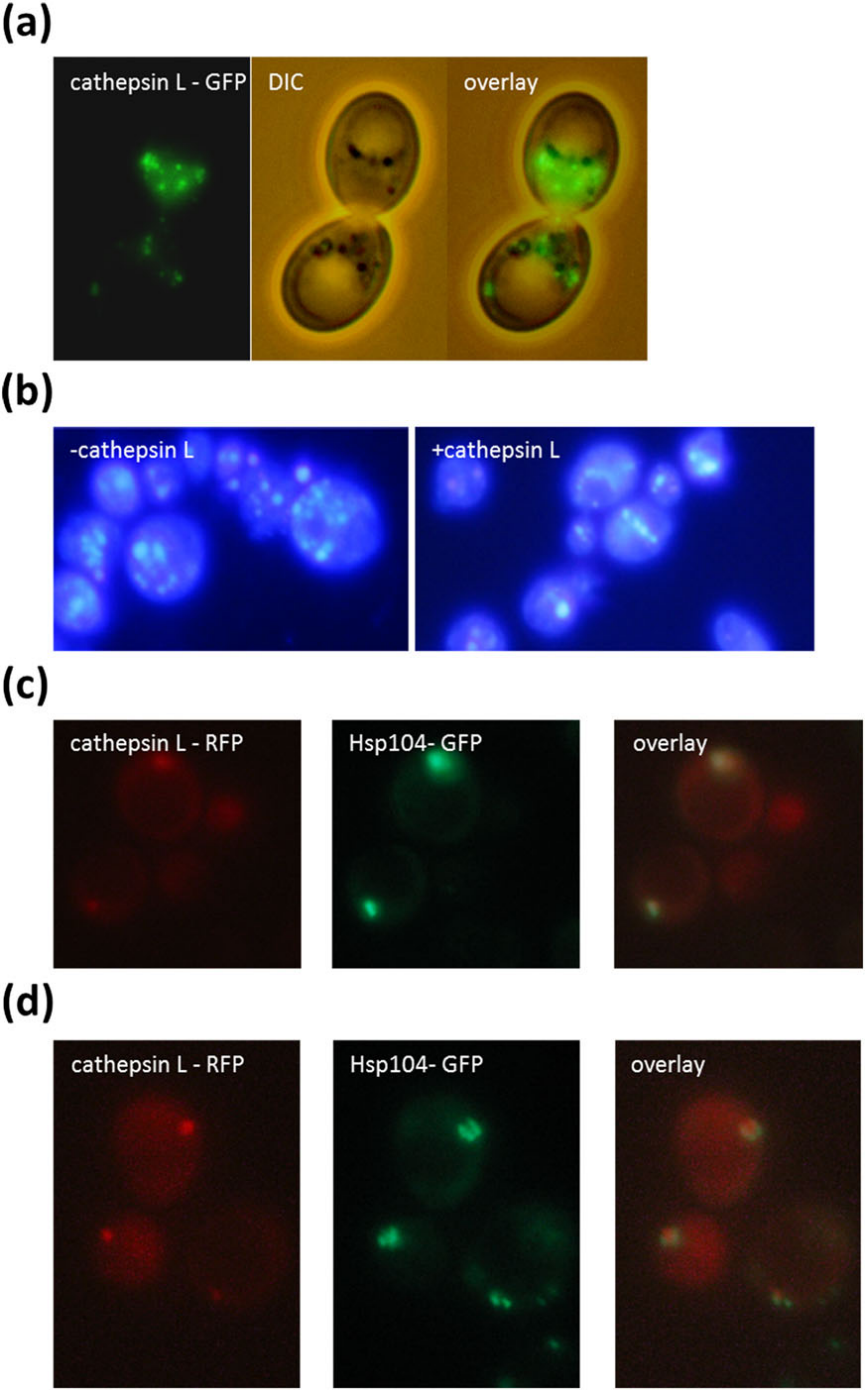
Localization of cathepsin L. GFP‐tagged Cathepsin L forms clear cytosolic foci in the strain BY4741 (BY4741 pUG35‐cathepsin L) (a). In the strain BY4741 p416GPD‐cathepsin L the number of protein aggregates stained BY the dye Thioflavin S is reduced compared with the strain BY4741 p416GPD (b). In the strain BY4741 HSP104‐GFP::HIS3 p416GPD‐cathepsin L‐RFP either a co‐localization between Hsp104‐GFP and cathepsin L‐RFP was detected (c) or it was observed that Hsp104‐GFP surrounds cathepsin L‐RFP (d)

In a first attempt to characterize cathepsin L a GFP tagged version was created by cloning its open reading frame (ORF) into the vector pUG35. Fluorescence microscopy of the strain BY4741 pUG35‐cathepsin L revealed a cytosolic punctate pattern (Figure 6a). These patterns resemble protein aggregates.

One of the hallmarks of replicative aging is the formation of protein aggregates and these aggregates are one of the main factors that shorten the lifespan of yeast cells. As has been shown previously, these aggregates are already formed early in life and are not passed on to daughter cells but are retained in the mothers (Saarikangas & Barral, 2015). Getting rid of these aggregates would be beneficial and this could explain the life‐prolonging effect of cathepsin L. Therefore protein aggregates (especially amyloid like proteins) were stained with the dye Thioflavin S (Kimura et al., 2003). As can be seen in Figure 4(b), a strain overexpressing *Dugesia* cathepsin L shows a clear reduction in the number of protein aggregates. One of the proteins that are prone to forming aggregates and limiting the lifespan is the yeast prion Sup35. In fact 20 cathepsin L cleavage sites could be predicted in Sup35, one with a specificity of more than 99.9% (amino acid 429–434: NFLR↓AI; http://www.dmbr.ugent.be/prx/bioit2‐public/SitePrediction/).

**Figure 7:**
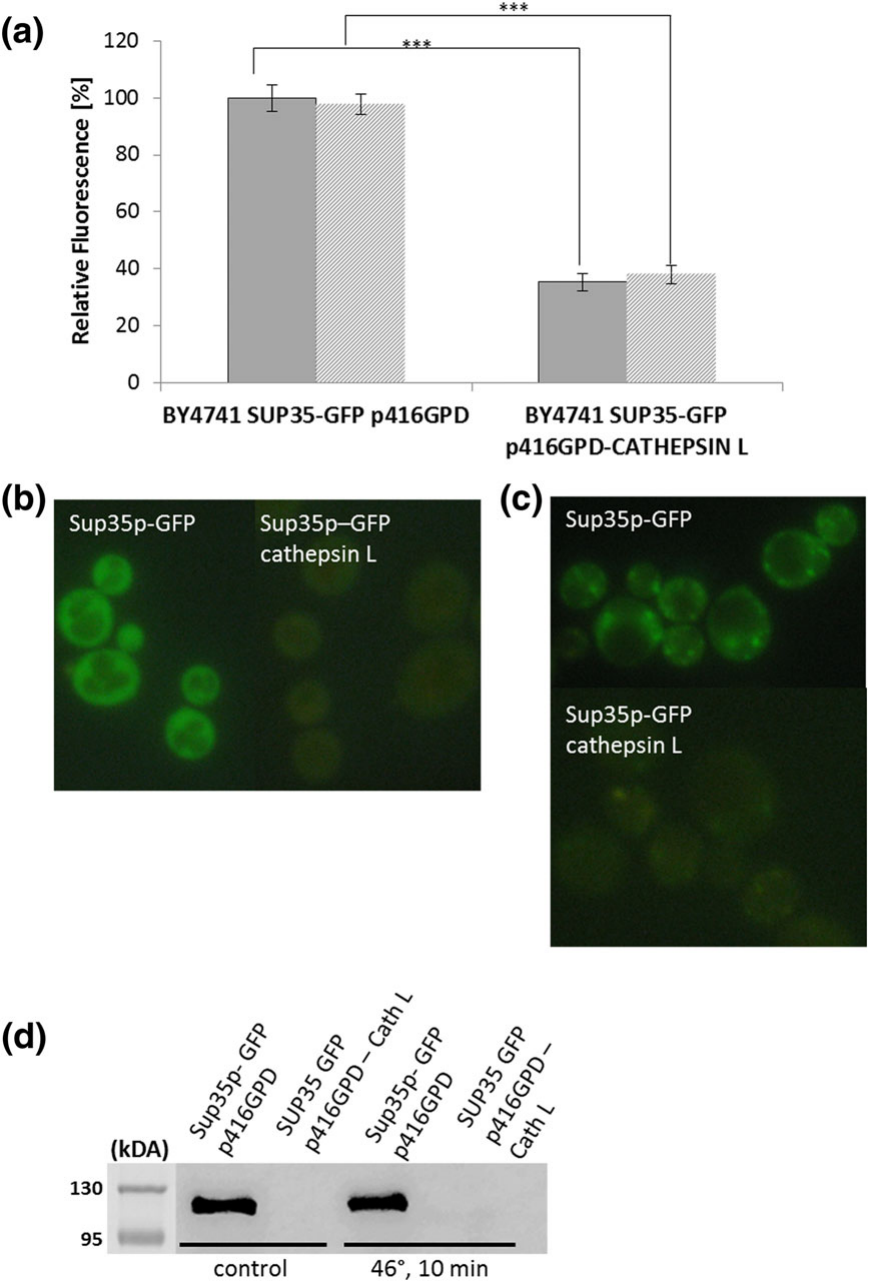
Sup35p and cathepsin L. Fluorometric measurements of the strains BY4741 SUP35‐GFP::HIS3 p416GPD and BY4741 SUP35‐GFP::HIS3 p416GPD‐cathepsin L that are either stressed (46°C) or unstressed. Expression of cathepsin L clearly and significantly (student *t*‐test; p‐value = 0.0001) decreases the Sup35p‐GFP fluorescence (a). Fluorescence microscopy of the same strains as in (a). Sup35‐GFP is equally distributed in the cytosol. Co‐expression of cathepsin L only leads to a very faint GFP‐signal (b). Fluorescence microscopy of the same strains as in (a) after a heat shock (46°C; 10 min) was applied. Sup35‐GFP clearly accumulates in cytosolic foci. These foci are nearly absent when cathepsin L is expressed. Western blot analysis of the same strains as in (a) with and without stress (46°C; 10 min) using an anti‐GFP antibody. In the control strain Sup35‐GFP is clearly detectable (with and without stress), whereas in the cathepsin L expressing strain only marginal protein levels are detectable [Colour figure can be viewed at wileyonlinelibrary.com]

A strain harbouring genomic integrated *SUP35*‐GFP obtained from the yeast GFP clone collection (ThermoFisher Scientific Waltham, MA, USA; Huh et al., 2003) was transformed with either the plasmid p416GPD or p416GPD‐cathepsin L. This vector leads to a constitutive expression of the endoprotease via the GPD (glyceraldehyde‐3‐phosphate dehydrogenase) promoter. The amount of GFP fluorescence was quantified using a fluorimeter. A clear decrease in fluorescence (more than 50%) was detected after expression of cathepsin L (see Figure 7a). We assume that cathepsin L leads to a fragmentation of Sup35. This effect seems to be specific for Sup35, because other proteins that are known to form protein aggregates were tested and these proteins (Ssa2‐GFP, Vma2‐GFP and Hsp104‐GFP) showed no such decrease in GFP fluorescence after cathepsin L expression (data not shown). However, fluorescence microscopy revealed that cathepsin L expression changes the distribution of Hsp104‐GFP. The disaggregating chaperone Hsp104 is believed to dissolve protein aggregates consisting of denatured proteins. There is also a certain risk that Hsp104 by breaking long amyloid filaments creates new foci on which prions can form (Cox, Byrne, & Tuite, 2007; Kryndushkin, Engel, Edskes, & Wickner, 2011). In the control strain (BY47141 p416GPD) Hsp104‐GFP can be found in the cytosol as well as in certain distinct cytosolic foci. Overexpression of cathepsin L (p146GPD‐cathepsin L) had no effect on the protein abundance of Hsp104‐GFP, but led to a disappearance of these Hsp104‐GFP foci in ~60% of all cells (Figure S2). In a follow‐up experiment we tried to co‐localize cathepsin L with Hsp104, which is a marker of protein aggregates. Therefore an RFP tagged version was created (p416GPD‐cathepsin L‐RFP) and was transformed into the yeast strain BY4741 HSP104‐GFP::HIS3. It has to be stated that in most cells either a GFP or a RFP signal in cytosolic foci was detected. In cells expressing both (Hsp104‐GFP and cathepsin L‐RFP) a co‐localization was observed with a relatively high Pearson's correlation coefficient (*Rr* = 0.6; Figure 6c). In certain cases it even looks as though the Hsp104‐GFP signal is surrounding the cathepsin L‐RFP signal (Figure 6d).

In the case of SUP35‐GFP the strain harbouring the empty plasmid showed a bright cytosolic signal (Figure 7b), whereas an expression of cathepsin L led to a very faint (if any) GFP signal. As we have shown previously (Rinnerthaler et al., 2013), a robust heat shock (10 min, 46°C) induces the formation of protein aggregates. In case of Sup35 protein aggregates became visible (Figure 7c). In contrast to this finding, a strain expressing cathepsin L showed only very faint yellowish protein aggregates and the overall GFP fluorescence was dramatically reduced (Figure 7a). Western blot analysis also revealed that Sup35‐GFP is nearly undetectable in a yeast cells that are expressing cathepsin L (in unstressed as well as heat shocked cells; Figure 7d). The cell tries to compensate for the loss of this essential protein by increasing the transcription of Sup35. RT‐PCR analysis revealed that in the strain BY4741 p416GPD‐cathepsin L the levels of SUP35 are significantly increased compared with the strain BY4741 p416GPD (3.5 ± 0.5‐fold; *p* = 0.05). This finding was also confirmed for aging. Young and old cells were obtained via elutriation. In young (fraction II) cells Sup35‐GFP was equally distributed in the cytosol, whereas in old cells (fraction V) clear Sup35‐GFP aggregates were observed. Overexpression of cathepsin L led to a complete disappearance of Sup35‐GFP from young and old cells (Figure S3). These experiments demonstrate that cathepsin L degrades Sup35, even before a stress occurs. These reduced levels of Sup35 in cells expressing cathepsin L do not result in the formation of aggregates anymore upon stress and aging. Of course we cannot rule out the possibility that some aggregates are left, but at an undetectable level. Therefore also fewer aggregates are stained by Hsp104‐GFP or Thioflavin S.

**Figure 8:**
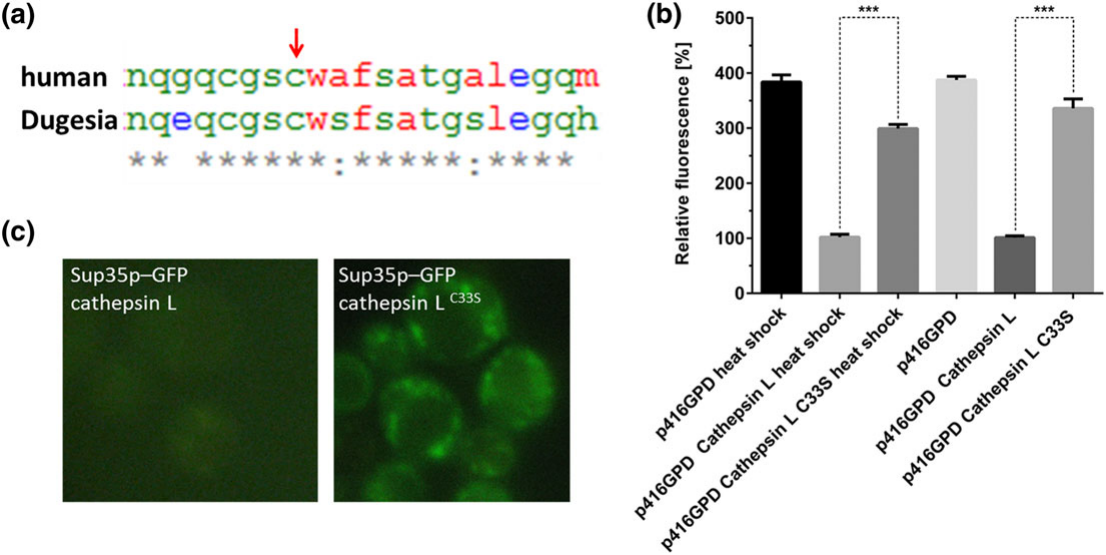
Inactivating mutant of cathepsin L. the detail of a sequence alignment between cathepsin L from Homo sapiens and D. tigrina is presented. The high degree of similarity and the conservation of the catalytic cysteine (indicated by an arrow) is obvious (a). Fluorimetric measurements of the strains BY4741 SUP35‐GFP::HIS3 p416GPD, BY4741 SUP35‐GFP::HIS3 p416GPD‐cathepsin L and BY4741 SUP35‐GFP::HIS3 p416GPD‐cathepsin L^C33S^ that are either stressed (46°C) or unstressed. Expression of cathepsin L^C33S^ does not significantly decrease the fluorescence intensity of Sup35p‐GFP, whereas the differences between cathepsin L and cathepsin L^C33S^ are highly significant (student *t*‐test; *p*‐value = 0.0001) (b). Fluorescence microscopy of the same strains as in (b) after heat stress (10 min 46°C). Expression of cathepsin L results in a not detectable GFP signal. Expression of cathepsin L^C33S^ leads to clearly visible Sup35‐GFP aggregates (c) [Colour figure can be viewed at wileyonlinelibrary.com]

In the next step we wanted to confirm that the loss of Sup35 can be attributed to the proteolytic activity of cathepsin L. Via a sequence alignment with human cathepsin L (Figure 8a), we identified the essential cysteine and replaced it with a serine (C33S). In fact the strain Sup35‐GFP harbouring the mutated plasmid (p416GPD‐cathepsin L^C33S^) showed no decrease in fluorescence intensity (Figure 8b) and in this strain clear Sup35‐GFP aggregates were visible after a 10 min heat stress at 46°C (Figure 8c).

### 3.5 Cathepsin L and *α*‐synculein

**Figure 9:**
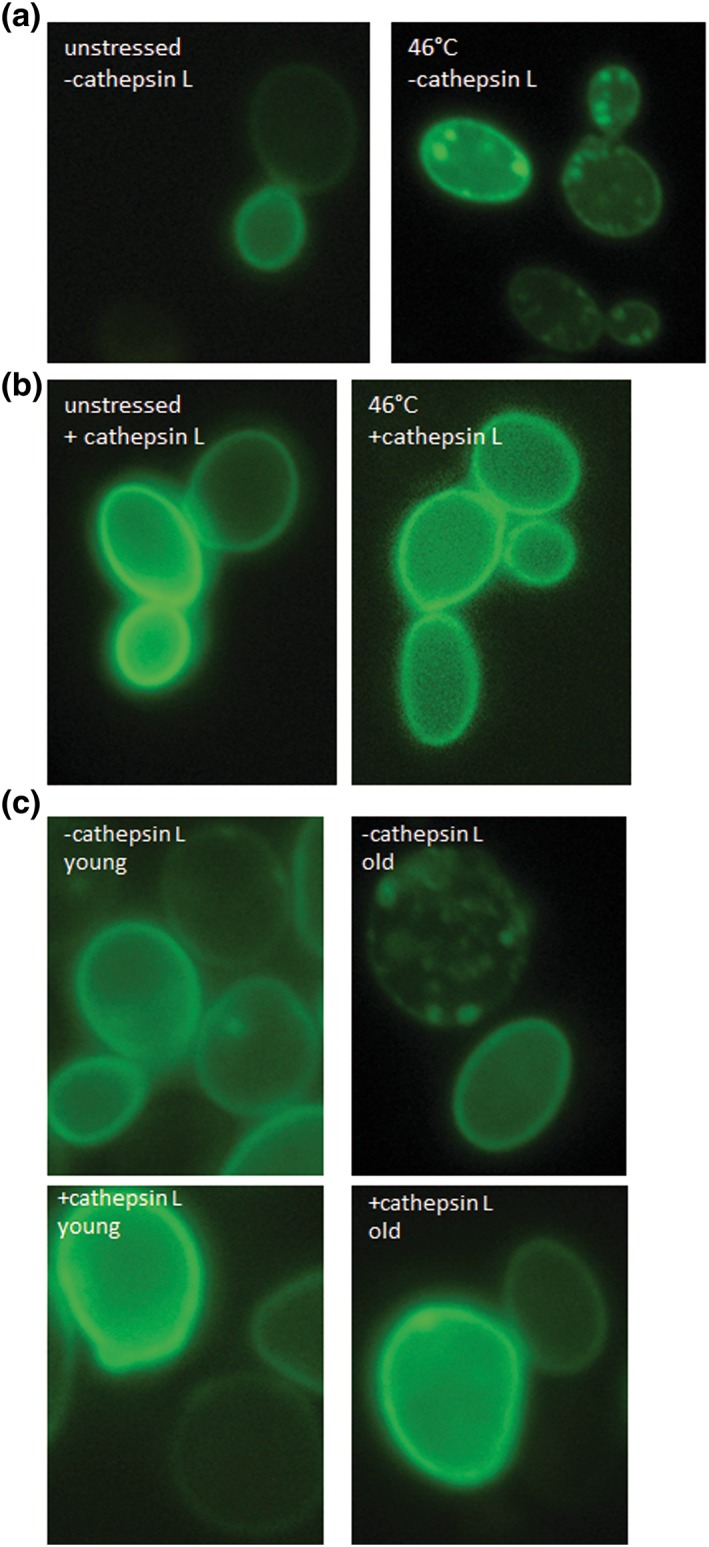
*α*‐Synuclein and cathepsin L. fluorescence microscopy of the strain BY4741 pUG35‐*α*‐synuclein pESC‐HIS. In unstressed cells *α*‐synuclein‐GFP is closely associated with the plasma membrane. A heat shock (10 min; 46°C) leads to a detachment of *α*‐synuclein from the plasma membrane and to an accumulation in cytosolic foci (a). Fluorescence microscopy of the strain BY4741 pUG35‐*α*‐synuclein pESC‐HIS cathepsin L. similar to (a) *α*‐synuclein‐GFP is localized at the plasma membrane but forms no visible cytosolic aggregates (b). Fluorescence microscopy of the strains BY4741 pUG35‐*α*‐synuclein pESC‐HIS and BY4741 pUG35‐*α*‐synuclein pESC‐HIS cathepsin L after elutriation. With and without the expression of cathepsin L *α*‐synuclein‐GFP is localized at the plama membrane in young cells (fraction II). In old cells (fraction V) *α*‐synuclein‐GFP detaches from the plasma membrane and forms aggregates. No such aggregates can be seen after expression of cathepsin L and the protein is still visible in the plasma membrane [Colour figure can be viewed at wileyonlinelibrary.com]

Protein aggregates do not only form upon stress, during aging and in the case of prion like proteins, but are also a hallmark of neurodegenerative diseases. In fact yeast was successfully established as a model organism to study Parkinson disease, one of the most common human neurodegenerative diseases (Petroi et al., 2012). It is believed that Parkinson disease is, among other things, the result of the formation of misfolded *α*‐synuclein aggregates that are termed Lewy bodies. A yeast strain expressing GFP‐tagged human *α*‐synuclein (BY4741 pUG35‐*α*‐synculein, pESC‐HIS) showed a clear plasma membrane localization (Figure 9a). Upon a 10 min 46°C heat shock *α*‐synuclein detaches from the plasma membrane and forms obvious cytosolic aggregates (Figure 9a). Co‐transformation with cathepsin L (BY4741 pUG35‐*α*‐synculein pESC‐HIS‐cathepsin L) leads to a complete disappearance of *α*‐synculein from the cytosol, whereas this human protein is still present in the plasma membrane (Figure 9b). In aged cells obtained via elutriation, *α*‐synculein detaches from the plasma membrane and is present in cytosolic foci. Overexpression of cathepsin L also completely removes these aggregates (Figure 9c).

## 4 CONCLUSION

In this study we demonstrated the feasibility of S. cerevisiae to screen for longevity genes. As a test candidate the planarian flatworm D. tigrina was chosen. By stimulating its regenerative capacity by beheading, this mortal plathelminth becomes potentially immortal. As the most probable candidate for this immortality, the enzyme telomerase is discussed in the literature (Tan et al., 2012). In animals the telomerase enzyme is mainly active in the germ line, but asexual forms of the flatworms can activate the telomerase after amputation, leading to a high somatic telomerase activity. To screen a cDNA library of D. tigrina for longevity genes, we successfully created an aging reporter in *S.cerevisiae*. Of all ~20,000 genes we found only one that significantly increased the replicative lifespan. This enzyme is not the ‘immortality telomerase’, but a cysteine protease: cathepsin L. To our knowledge this enzyme was sequenced for the first time in this study. An overexpression of D. tigrina cathepsin L led to a 34% increase in the replicative lifespan of yeast cells.

Among the animal kingdom there are already hints that cathepsin L could be a longevity gene. In certain tumours cathepsin L is massively upregulated, thus promoting the malignancy of tumours. One reason to induce the expression of cathepsin L is very obvious. Secretion of cathepsin L by tumour cells leads to a degradation of extracellular matrix components such as collagen, fibronectin and laminin and enables the migration of tumour cells. However, it has also been demonstrated that cathepsin L inhibits apoptosis by stimulating the expression of Bcl‐2 (Levicar et al., 2000). The main regulator of cathepsin L expression is the human transcription factor FOXO3a. The importance of FOXO3a for the aging process has already been demonstrated. For example the effect of dietry restriction seems to be closely related to the FOXO transcription factors. This was first shown in C. elegans. In this organism the nutrient sensing pathway upon its activation by the receptor DAF‐2 leads to a phosphorylation and inactivation of the effector protein DAF‐16, which is a fork‐head box O (FOXO) transcription factor. Inactivating mutants in daf‐2 leads to a translocation of DAF‐16 to the nucleus and to the transcription of a large group of genes that are involved in stress response, fat metabolism, cellular protection and dauer formation (Tullet, 2015). This FOXO transcription factor is highly conserved and can be found in humans and *Hydra* with some spectacular aging phenotypes.

*Hydra*, a freshwater polyp, is probably one of the rare organisms that are potentially immortal. Loss of foxO in the immortal *Hydra* increases the number of differentiated cells and decreases the number of stem cells, thus limiting the lifespan of this polyp (Boehm et al., 2012). Furthermore, single nucleotide polymorphisms (SNPs) in the human transcription factor FOXO3A can increase the probability of an increased lifespan, because different SNPs were found in people >100 years old; Flachsbart et al., 2009). We have shown that the closest homologue to cathepsin L from D. tigrina can be found in *Hydra vulgaris*. We were also able to predict FOXO response elements in the promotor of cathepsin L using JASPAR (http://jaspar.genereg.net/cgi‐bin/jaspar_db.pl). It would be thrilling to see if a knock down of cathepsin L would limit the lifespan of H. vulgaris. In yeast cells we have been able to demonstrate that cathepsin L reduces the levels of the prion protein Sup35, thus limiting the formation of protein aggregates, one of the hallmarks of aging. This effect was also confirmed with Thioflavin S staining. After cathepsin L expression fewer protein aggregates were observed, although they were not completely removed. We could also demonstrate that cathepsin localizes to the same protein aggregates as the disaggregase Hsp104. Additionally we could show that overexpression of cathepsin L stimulates autophagy. Both reduction of protein aggregates and stimulation of autophagy are most probably how the increase in replicative lifespan is achieved after overexpression of cathepsin L. Our data also clearly indicate that cathepsin L from D. tigrina has the ability to dissolve Lewy bodies consisting of aggregated human *α*‐synuclein molecules in yeast cells. It seems to us that cathepsin L recognizes and degrades unstructured proteins such as *α*‐synuclein and SUP35, but has no effect on such proteins as Hsp104.

## CONFLICT OF INTEREST

The authors confirm no conflict of interest.

## Supporting information

Figure S1 Supporting info item
