# Supplementary figures and images for: Behead and live long or the tale of cathepsin L
Source: Yeast. 2017 Nov 29;35(2):237–49. doi: 10.1002/yea.3286 (PMC5808862; doi:10.1002/yea.3286)

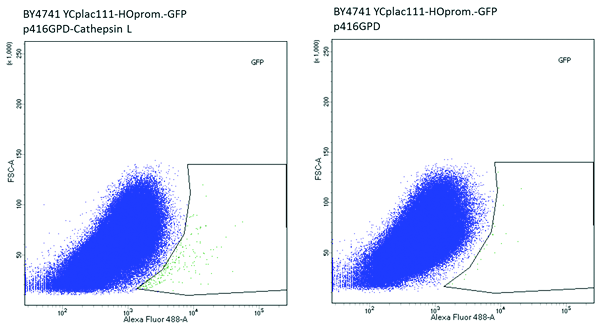

Supplement: Supplementary file 1 — Figure S1 Supporting info item [file YEA-35-237-s001.zip › Supp 1.tif]

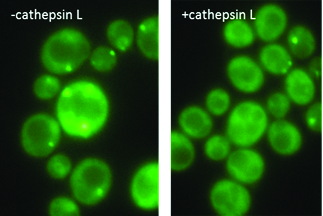

Supplement: Supplementary file 1 — Figure S1 Supporting info item [file YEA-35-237-s001.zip › Supp 2.tif]

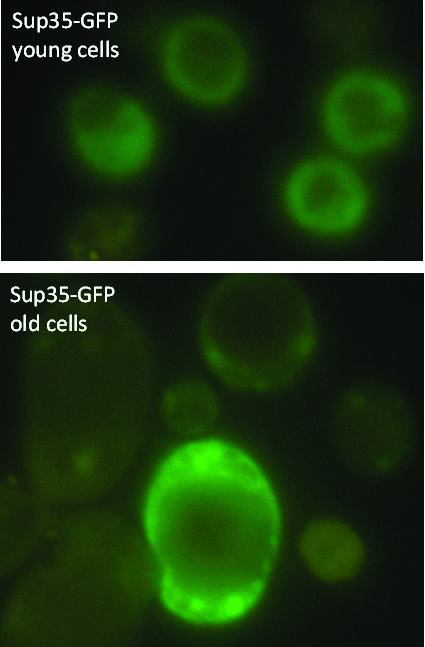

Supplement: Supplementary file 1 — Figure S1 Supporting info item [file YEA-35-237-s001.zip › Supp 3.tif]
